# Illicit stimulants and ventricular arrhythmias: a longitudinal cohort study

**DOI:** 10.1093/eurheartj/ehaf282

**Published:** 2025-05-07

**Authors:** Jean Jacques Noubiap, Thomas A Dewland, Gabrielle C Montenegro, Hannah H Oo, Zian H Tseng, Gregory M Marcus

**Affiliations:** Division of Cardiology, Department of Medicine, University of California-San Francisco, San Francisco, CA, USA; Division of Cardiology, Department of Medicine, University of California-San Francisco, San Francisco, CA, USA; Division of Cardiology, Department of Medicine, University of California-San Francisco, San Francisco, CA, USA; Division of Cardiology, Department of Medicine, University of California-San Francisco, San Francisco, CA, USA; Division of Cardiology, Department of Medicine, University of California-San Francisco, San Francisco, CA, USA; Division of Cardiology, Department of Medicine, University of California-San Francisco, San Francisco, CA, USA

**Keywords:** Cocaine, Methamphetamine, Ventricular tachycardia, Ventricular fibrillation, Cardiac arrest, Death

## Abstract

**Background and Aims:**

Ventricular tachycardia and ventricular fibrillation underlie many sudden cardiac deaths, but common lifestyle factors that predict their occurrence are poorly understood. This study aimed to assess the association between methamphetamine and cocaine, the most used illicit stimulants, and ventricular arrhythmias (VA) and mortality.

**Methods:**

Healthcare databases were used to identify adults aged ≥ 18 years receiving hospital-based care in California in 2005–2019. ICD codes were used to identify diagnoses and illicit stimulant use. Cox proportional hazard models adjusting for demographics and time-updated cardiovascular risk factors were employed. The outcomes were VA combining ventricular tachycardia, ventricular fibrillation, and cardiac arrest and all-cause mortality.

**Results:**

Among 29 593 819 individuals (53.8% female, mean age 44.9 years), 690 737 (2.3%) used methamphetamine, and 290 652 (1.0%) used cocaine at some point. After adjustment for age, sex, race and ethnicity, cannabis and opioid use, and other cardiovascular risk factors, methamphetamine use and cocaine use were each associated with increased risk of incident VA [hazard ratio (HR) 1.90, 95% confidence interval (CI) 1.85–1.95, and HR 1.15, 95% CI 1.10–1.19, respectively], and mortality (HR 1.51, 95% CI 1.47–1.54 and HR 1.68, 95% CI 1.64–1.72, respectively). The risk of VA was higher in younger individuals (<65 years) and females for both methamphetamine and cocaine, whereas it was higher in Blacks and lower in Whites and Hispanics for cocaine use, and higher in Asians and lower in Blacks for methamphetamine use (*P* for interaction < .05 in all).

**Conclusions:**

Methamphetamine use and cocaine use were each associated with increased risk of incident VA and mortality, with differential relationships by demographic groups. Avoiding these substances may reduce risk of VA and death.

## Structured Graphical Abstract

**Structured Graphical Abstract:**
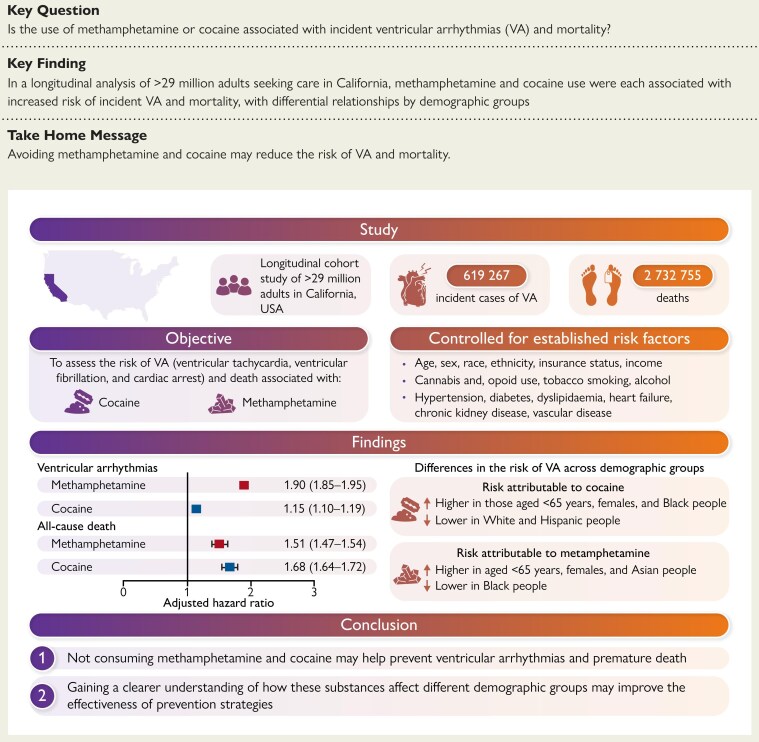

**See the editorial comment for this article ‘Illicit stimulants and sudden cardiac death: fatal attraction and sex interaction’, by O. Weizman and E. Marijon, https://doi.org/10.1093/eurheartj/ehaf443.**

## Introduction

Sustained ventricular arrhythmias (VA), including ventricular tachycardia (VT) and ventricular fibrillation (VF), are an important cause of morbidity and a common cause of sudden cardiac death (SCD),^1,2^ accounting for many of the approximate 300 000 cases occurring in the United States every year.^1^ Several effective therapies for the management of VA and prevention of SCD have been developed, including antiarrhythmic medications, surgical and catheter ablation, autonomic modulation, implantable cardiac defibrillators (ICDs), and revascularization therapies for ischaemic heart disease.^1,2^ These approaches are notably either for secondary prevention for those who have already suffered from these arrhythmias or they treat, rather than prevent, the adverse consequences (such as primary prevention ICDs). Nevertheless, unlike other heart diseases, such as atrial fibrillation,^3^ true primordial prevention through lifestyle modification is still not a major focus in VA.

Illicit drug use is a growing global health problem. In 2018, about 269 million people misused drugs for nonmedical purposes, representing 5.3% of people aged 15–64 years worldwide,^4^ and occult overdose underlies 1 in 6 presumed SCDs.^5^ Amphetamines and cocaine are the most used stimulants for nonmedical purposes.^4^ Although some studies have suggested a link between methamphetamine and cocaine and VA and SCD, the quality of evidence is relatively low.^6^ Indeed, most of these studies were of small sample size^7–11^ or case reports.^12–15^ Many were conducted in very selected populations such as those with hypertension,^9^ heart failure,^16^ myocarditis,^7^ chest pain,^10^ or established ischaemic heart disease,^8^ limiting the extrapolation of their findings to the general population. Furthermore, a large proportion of studies were cross-sectional,^16–22^ including post-mortem toxicologic analysis of cases of SCD.^17,18,20–22^ In some of these SCD autopsy series, a significant proportion of methamphetamine or cocaine users exhibited non-toxic levels of the drug^21,22^ or also had other substances detected,^20,22^ making the direct role of methamphetamine or cocaine in these SCD cases less certain.

Therefore, whether and to what extent these commonly used illicit stimulants lead to VA and death remains unclear. Given this limited understanding, we performed a population-based, longitudinal cohort study examining methamphetamine and cocaine uses as predictors of incident VT, VF, cardiac arrest (CA), and all-cause mortality.

## Methods

### Data source, study design, and population

The California's Department of Health Care Access and Information (HCAI) databases were used to identify all individuals aged ≥ 18 years who received care in a California emergency department, ambulatory surgery unit, or inpatient hospital between 1 January 2005 and 31 December 2019. To capture repeated healthcare encounters for a given patient and follow them across multiple encounters over time, we merged individual databases specific to healthcare settings (emergency department, ambulatory surgery unit, and inpatient hospital) using each patient's record linkage number, a unique 9-digit alphanumeric value that is the encrypted form of a patient's Social Security Number. Healthcare encounters with missing data regarding age, sex, and race and ethnicity were excluded. Death information was retrieved from the HCAI healthcare databases and from the California Comprehensive Death File.

### Diagnosis ascertainment

Diagnoses were identified through the International Classification of Diseases, Ninth Revision (ICD-9) code from January 2005 to September 2015 and the International Classification of Diseases, Tenth Revision (ICD-10) codes from October 2015 to December 2019 (see Supplementary data online, *Table S1*). In addition to ICD-9 and ICD-10 codes, we used the Clinical Classification Software codes for diseases with specific Clinical Classification Software codes (see Supplementary data online, *Table S1*). Up to 25 ICD codes and 25 Clinical Classification Software codes were recorded for each healthcare encounter.

### Study variables and outcomes

#### Exposure

Substance use (methamphetamine, cocaine, cannabis, and opioid) was ascertained based on ICD-9 and ICD-10 codes (either primary or secondary diagnoses) from the emergency department, ambulatory surgery unit, or inpatient hospital. The main exposures were methamphetamine use and cocaine use. The validity of ICD codes for the identification of methamphetamine use and cocaine use was assessed for each illicit stimulant use in 300 patients (100 patients with at least one ICD code for amphetamine use, 100 with a code for cocaine use, and 100 patients without any codes for stimulant use) seen at the University of California, San Francisco Medical Center during the study period. We then reviewed the electronic health records of these patients to determine whether a clinical note confirmed the use of methamphetamine or cocaine.

#### Covariates

We also collected information on patients’ age, sex, race and ethnicity, insurance status, income level, and selected chronic diseases. We used the normalized race group for a patient based on a combination of their reported race and ethnicity. Hispanic ethnicity was treated as a distinct group that superseded the race so that individuals with Hispanic ethnicity were coded as Hispanic, and for other individuals, the normalized race group was assigned the same value as the reported race. Therefore, race and ethnicity were mutually exclusive and categorized as Asian/Pacific Islander, Black, Hispanic, Native American (American Indian/Alaska Native), White, and ‘Other’. Substance use (methamphetamine, cocaine, cannabis, and opioid) was ascertained based on ICD-9 and ICD-10 codes (either primary or secondary diagnoses) from the emergency department, ambulatory surgery unit, or inpatient hospital. Income level was estimated using the median household income for the patient's ZIP code. Insurance status was categorized as Medicare, Medicaid, private insurance, self-pay, and others. Co-morbidities, tobacco smoking, and alcohol abuse were assessed and updated at each healthcare encounter for every participant, and once they were present, they were carried forward over time. For instance, once a diagnosis of hypertension was made, the participant was considered as having hypertension at all subsequent healthcare encounters. Substance use, income level, and insurance status were determined at each healthcare encounter and defined independently of previous encounters, serving as time-varying variables.

#### Outcomes

The outcomes were incident VT, VF, CA, and all-cause mortality.

### Statistical analysis

Categorical variables are expressed as frequencies and percentages while continuous variables are expressed as means with standard deviations. We assessed differences between groups using the Student's *t*-test for continuous variables and the Pearson Chi-square test for categorical variables. Times to incident VT, VF, CA, VA (combining VT, VF, and CA), and all-cause mortality were assessed using survival analyses. Patients entered the cohort at their first healthcare encounter and were censored when they reached a study outcome for a given analysis, at death, or at the end of follow-up (31 December 2019). Patients with an outcome at the first recorded healthcare encounter were excluded from the specific outcome analysis but contributed to the other outcome analyses. For instance, a patient with VT at first encounter was excluded from the VT survival analysis but could contribute to the VF, CA, or all-cause mortality survival analyses. Death was considered as a competing risk for the outcomes of VT, VF, CA, and VA. We used multivariable Cox regression analysis to assess the association between time-varying methamphetamine and cocaine use and incident outcomes, with adjustment for potential confounders and mediators selected *a priori* as plausible confounders and mediators of the outcomes based on biological plausibility and the medical literature. These included age, sex, race and ethnicity, hypertension, diabetes mellitus, heart failure, dyslipidaemia, chronic kidney disease, coronary artery disease, peripheral artery disease, ischaemic stroke, alcohol abuse, tobacco smoking, opioid and cannabis uses, insurance status, and income level. Interaction testing by sex, age (dichotomized by 65 years), race and ethnicity, and income level (dichotomized by median income) was also performed, with methamphetamine and cocaine uses as the predictors, and VA as the outcome. Because human immunodeficiency virus (HIV) is known to be associated with VA and users of these stimulants might be more likely to harbour HIV,^23–25^ we performed a sensitivity analysis excluding patients with HIV. Furthermore, as amphetamine is a treatment option for patients with obesity-related disorders, and attention-deficit/hyperactivity disorder (ADHD) and narcolepsy, we also performed sensitivity analyses excluding patients with these conditions.

To better capture cases of out-of-hospital cardiac arrest, we performed a sensitivity analysis focusing on CA coded in the emergency department only (supported by evidence that ICD coding for CA is accurate in identifying emergency department patients who suffer OHCA^26^). In addition, we performed a sensitivity analysis focusing on out-of-hospital total mortality in order to examine those who died outside the emergency department or hospital.

To assess the validity of our analyses and by examining for evidence of systemic bias that might differentially misclassify outcomes in favour of significant associations in general with those coded as using methamphetamine or cocaine, the main statistical analyses were repeated using presbycusis (age-related hearing loss), myasthenia gravis, and acromegaly (none of which were expected to be associated with substance use) as negative controls outcomes. While all Cox regression analyses were performed using time-updated covariates with the inclusion of repeated healthcare visits, the production of survival curves for illustrative purposes was not possible using the very large dataset of over 170 million observations due to feasibility constraints related to computing capacities. Therefore, to generate a survival curve figure for the combined outcome of VA, the follow-up of each participant was divided into two possible periods: (i) free of methamphetamine and cocaine use and (ii) cocaine use or methamphetamine use. A Cox regression analysis was then conducted with censoring for incident VA, death, or the end of follow-up (31 December 2019), and survival curves generated. Finally, to further validate our findings, we performed analyses using propensity score matching. Individuals with cocaine use and methamphetamine use were matched 1:1 at baseline (at the time of first substance use diagnosis) with individuals without cocaine use and methamphetamine use based on age, sex, race, and co-morbidities (hypertension, diabetes mellitus, heart failure, dyslipidaemia, chronic kidney disease, coronary artery disease, peripheral artery disease, chronic obstructive pulmonary disease, and ischaemic stroke). Then, we performed a Cox regression analysis using a single observation per participant to assess the effect of cocaine use and methamphetamine use on the time to incident VA. Risk estimates are reported as adjusted hazard ratios (HRs) with 95% confidence intervals (CIs). A two-tailed *P*-value of ≤ .05 was considered statistically significant.

To assess the accuracy of ICD codes to identify patients with methamphetamine and cocaine use, we calculated sensitivities and specificities with 95% CI using manual review of medical records as the reference standards. All analyses were performed using Stata 18 statistical package (StataCorp, College Station, TX, USA).

This study received approval from the Institutional Review Board of the University of California-San Francisco.

## Results

### Validity of diagnosis codes for illicit stimulant use

The ICD codes used had a sensitivity and a specificity of 97.0% (95% CI 91.5–99.4) for identifying methamphetamine use, and a sensitivity of 93.5% (87.0–97.3) and a specificity of 100% (96.1–100) for identifying cocaine use (see Supplementary data online, *Table S2*).

### Population characteristics

After applying exclusion criteria (see Supplementary data online, *Figure S1*), we included 29 593 819 patients experiencing 171 526 317 healthcare encounters over a median follow-up of 9.8 years (interquartile range 5.1–13.1 years). In total, 690 737 (2.3%) and 290 652 (1.0%) patients were identified at some point during the follow-up period as having diagnostic codes for methamphetamine use and cocaine use, respectively. Patient characteristics at first healthcare encounter, stratified by methamphetamine and cocaine uses, are shown in *Table 1*. A total of 314 040 developed incident VT, 72 278 developed incident VF, 333 788 experienced a CA, 619 267 experienced VA (VT, or VF, of CA), and 2 732 755 died.

**Table 1:** Participant characteristics, stratified by use of methamphetamine and cocaine at first healthcare encounter

Variables	No use of either substance (*n* = 29 409 682)	Methamphetamine use only (*n* = 119 247)	Cocaine use only (*n* = 54 289)	Use of both substances (*n* = 10 601)
Mean age (years)	45.1 ± 19.4	34.6 ± 12.4	38.7 ± 13.5	34.8 ± 12.6
Male (%)	13 536 252 (46.0)	82 109 (68.9)	39 602 (73.0)	8023 (75.7)
Race and ethnicity (%)				
Asian	2 923 003 (9.9)	4774 (4.0)	1634 (3.0)	372 (3.5)
Black	2 162 061 (7.4)	7366 (6.2)	16 901 (31.1)	1401 (13.2)
Hispanic	7 714 962 (26.3)	38 169 (32.0)	12 929 (23.8)	3091 (29.2)
Native American	105 551 (0.4)	779 (0.7)	139 (0.3)	36 (0.3)
White	15 218 107 (51.8)	63 749 (53.5)	20 860 (38.4)	5329 3 (50.3)
Other	1 285 998 (4.4)	4410 (3.7)	1826 (3.4)	372 (3.5)
Co-morbidities				
Diabetes (%)	2 033 332 (6.9)	6121 (5.1)	4040 (7.4)	609 (5.7)
Hypertension (%)	4 400 036 (15.0)	13 952 (11.7)	9990 (18.4)	1490 (14.1)
Heart failure (%)	462 326 (1.6)	4648 (3.9)	2407 (4.4)	509 (4.8)
Dyslipidemia (%)	2 115 882 (7.2)	4135 (3.5)	3078 (5.7)	570 (5.4)
Obesity-related disorders	769 533 (2.6)	3145 (2.6)	1669 (3.1)	366 (3.5)
CKD (%)	328 554 (1.1)	1187 (1.0)	1026 (1.9)	145 (1.4)
CAD (%)	1 013 763 (3.5)	3517 7 (3.0)	3041 (5.6)	510 (4.8)
PAD (%)	148 790 (0.5)	312 (0.3)	221 (0.4)	33 (0.3)
Ischaemic stroke (%)	208 483 (0.7)	1470 (1.2)	902 (1.7)	144 (1.4)
Narcolepsy	2099 (0.0)	21 (0.0)	12 (0.0)	<11 (0.0)^a^
ADHD	50 838 (0.2)	1374 (1.2)	508 (0.9)	205 (1.9)
HIV infection	21 505 (0.1)	981 (0.8)	575 (1.1)	134 (1.26)
Other substance use				
Cannabis (%)	166 412 (0.6)	20 877 (17.5)	10 660 (19.6)	3659 (34.5)
Opioid (%)	77 710 (0.3)	9281 (7.8)	6693 (12.3)	1880 (17.7)
Alcohol abuse (%)	618 241 (2.1)	21 641 (18.2)	18 409 (33.9)	3826 (36.1)
Tobacco smoking (%)	2 150 501 (7.3)	40 737 (34.2)	19 331 (35.6)	4469 (42.2)
Income quartile				
First (lowest)	5 876139 (20.0)	31 929 (26.8)	16 536 (30.5)	2612 (24.6)
Second	6 485 552 (22.1)	30 117 (25.3)	10 623 (19.6)	2289 (21.6)
Third	7 104 697 (24.2)	23 579 (19.8)	10 225 (18.8)	2069 (19.5)
Fourth	9 943 294 (33.8)	33 622 (28.2)	16 905 (31.1)	3631 (34.3)
Payer source				
Medicare	3 875 553 (13.2)	5083 (4.3)	3451 (6.4)	618 (5.8)
Medi-Cal	4 078 445 (13.9)	33 807 (28.4)	11 676 (21.5)	2407 (22.7)
Private	15 791 559 (53.7)	26 929 (22.6)	16 548 (30.5)	3206 (30.2)
Self-pay	3 738 798 (12.7)	37 657 (31.6)	14 533 (26.8)	2737 (25.8)
Others	1 925 327 (6.6)	15 771 (13.2)	8081 (14.9)	1633 (15.4)

ADHD, attention-deficit/hyperactivity disorder; CAD, coronary artery disease; CKD, chronic kidney disease; HIV, human immunodeficiency virus; PAD, peripheral artery disease.

^a^To protect the confidentiality of patients, no cell containing a value <11 can be reported directly.

### Risks of ventricular arrhythmias and mortality from stimulant use

**Figure 1:**
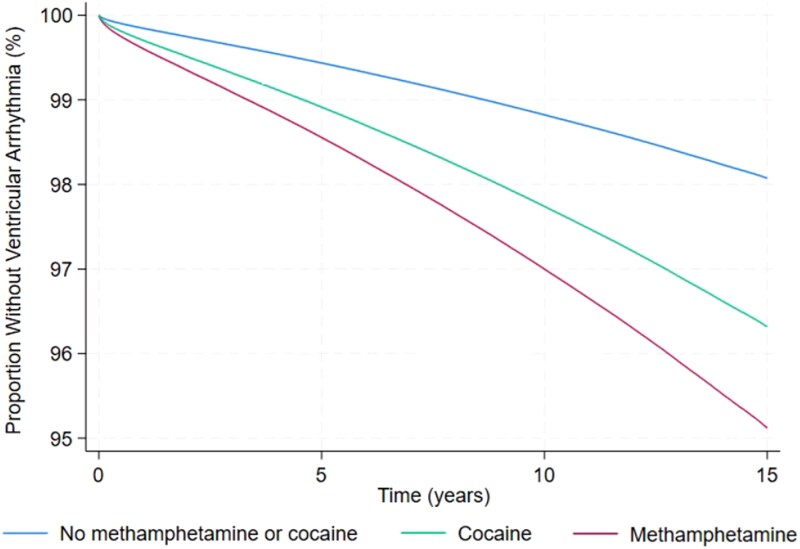
Multivariable adjusted survival curves for ventricular arrhythmias. Adjusted for demographics (age, sex, race, and ethnicity), insurance status, level of income, substance use (methamphetamine, cocaine, cannabis, and opioid), tobacco smoking, alcohol abuse, and co-morbidities (hypertension, diabetes, dyslipidaemia, heart failure, chronic kidney disease, coronary artery disease, peripheral artery disease, ischaemic stroke). Ventricular arrhythmias combine ventricular tachycardia, ventricular fibrillation, and cardiac arrest

**Figure 2:**
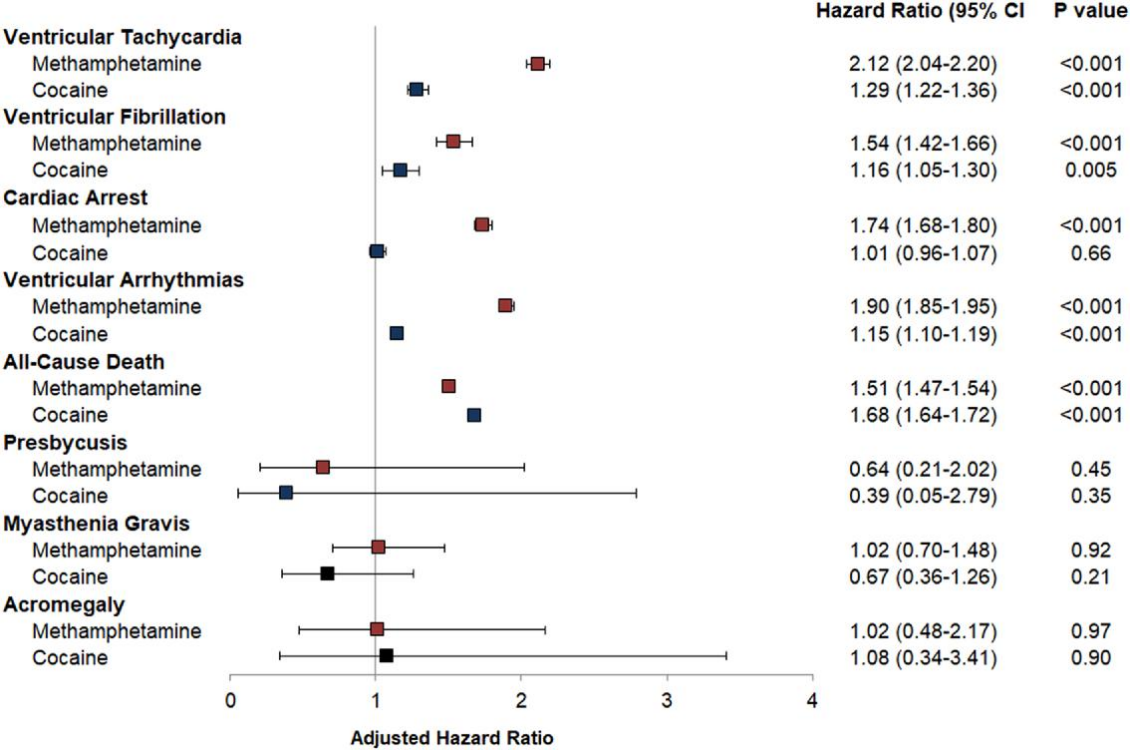
Adjusted risk of ventricular tachycardia, ventricular fibrillation, cardiac arrest, ventricular arrhythmia, all-cause mortality, presbycusis, myasthenia gravis, and acromegaly (negative controls) associated with methamphetamine and cocaine use. Hazard ratio with 95% confidence interval, adjusted for demographics (age, sex, race, and ethnicity), insurance status, level of income, substance use (methamphetamine, cocaine, cannabis, opioid), tobacco smoking, alcohol abuse, and co-morbidities (hypertension, diabetes, dyslipidaemia, heart failure, chronic kidney disease, coronary artery disease, peripheral artery disease, ischaemic stroke)

Individuals using methamphetamine or cocaine had higher crude incidence rates of VT, VF, CA, VA, and all-cause mortality (see Supplementary data online, *Table S3*). After adjusting for age, sex, race and ethnicity, payer source, income level, use of other substances, and co-morbidities, methamphetamine and cocaine uses were each associated with increased incidence of VT, VF, VA, all-cause mortality, and out of hospital mortality (*Figures 1* and *2*, and Supplementary data online, *Table S4*). The heightened risks of VA among those who used methamphetamine and cocaine persisted in sensitivity analyses excluding patients with HIV, obesity-related disorders, and ADHD and narcolepsy (see Supplementary data online, *Table S5*) and in the sensitivity analysis using propensity matched cohorts (see Supplementary data online, *Tables S6*–*S8*). Methamphetamine use, but not cocaine use, was associated with a higher incidence of CA (*Figure 2*), and specifically CA coded in emergency departments in a sensitivity analysis (see Supplementary data online, *Table S4*). There were no statistically significant associations between either methamphetamine use or cocaine use and the negative control outcomes, presbycusis, myasthenia gravis, and acromegaly (*Figure 2*).

**Figure 3:**
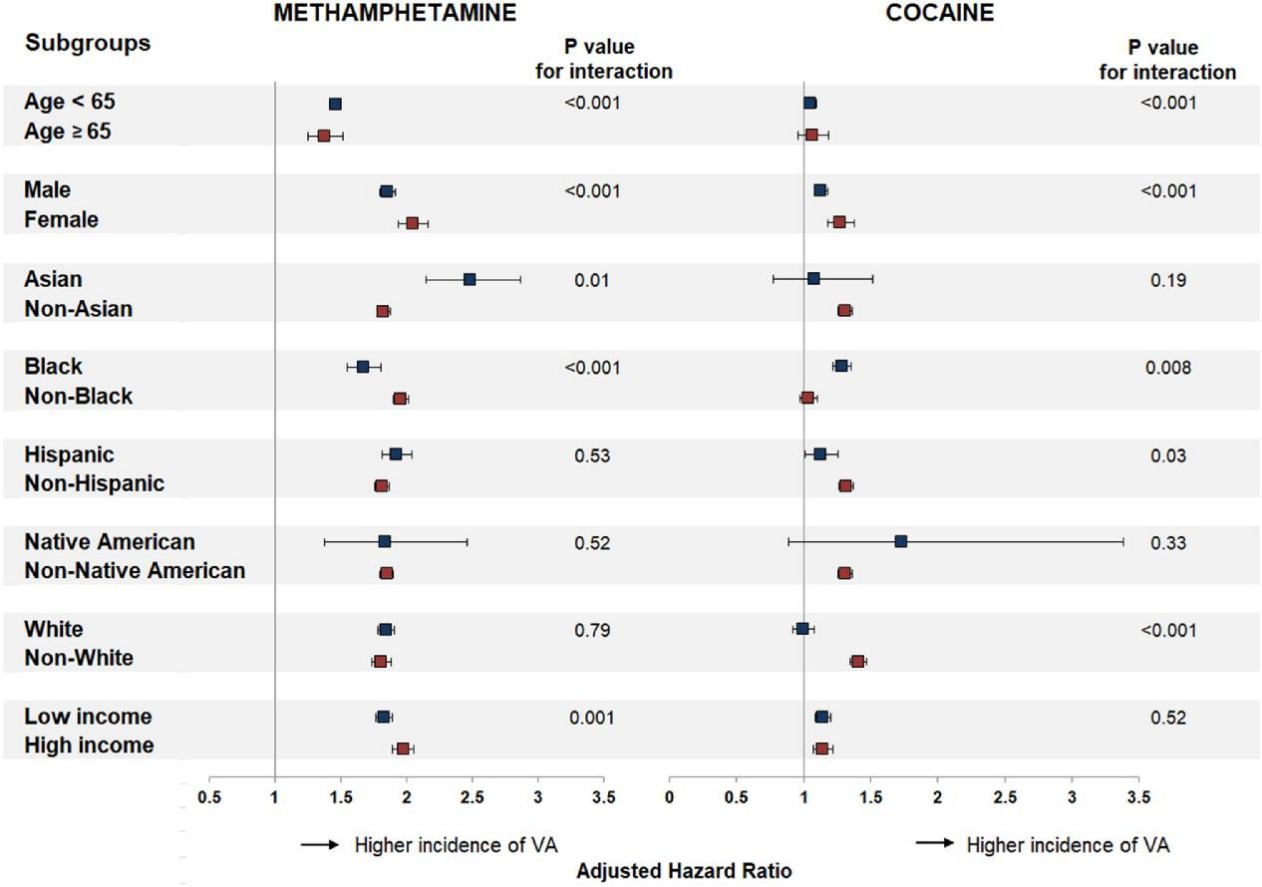
Adjusted risk of ventricular arrhythmias associated with methamphetamine and cocaine use across sociodemographic groups. Hazard ratio with 95% confidence interval, adjusted for demographics (age, sex, race, and ethnicity), insurance status, level of income, substance use (methamphetamine, cocaine, cannabis, opioid), tobacco smoking, alcohol abuse, and co-morbidities (hypertension, diabetes, dyslipidaemia, heart failure, chronic kidney disease, coronary artery disease, peripheral artery disease, ischaemic stroke). Ventricular arrhythmias combine ventricular tachycardia, ventricular fibrillation, and cardiac arrest. Comparators in race and ethnicity assessments include all patients not of that race and ethnicity

There were disparities in the effect of methamphetamine and cocaine uses on VA across sociodemographic groups. The relative risk of VA was higher in individuals aged < 65 years and females for both methamphetamine and cocaine. This risk was higher in Blacks and lower in Whites and Hispanics for cocaine use and higher in Asians and lower in Blacks for methamphetamine use (*Figure 3*). Whereas people with higher income tended to have a higher risk of VA associated with methamphetamine use, there was no interaction between cocaine use and the level of income.

## Discussion

Among this large population-based cohort of patients seeking care in California, methamphetamine and cocaine uses were each associated with increased incidence of VA and all-cause mortality, with magnitudes of risk that were comparable to those of several classic cardiovascular risk factors. Furthermore, the risk of VA varied across sociodemographic groups, with relative risks consistently higher in younger individuals and females for both methamphetamine and cocaine use, higher in Blacks and lower in Whites and Hispanics for cocaine use, and higher in Asians and lower in Blacks for methamphetamine use (*Structured Graphical Abstract*).

*In vivo* and *in vitro* studies in animal models and human case studies have shown that methamphetamine and cocaine induce electrical and structural changes that can initiate cardiac arrhythmias.^6^ Cocaine can produce electrical remodelling by altering gating properties of ion channels.^6^ Cocaine inhibits several potassium channels, including voltage-dependent potassium channels that prolong the QT interval.^10^ In addition, cocaine blocks fast inward sodium channels similar to class IC antiarrhythmic drugs^27^ and also alters the function of calcium-related channels and calcium handling proteins, resulting in a dose-dependent increase in action potential duration,^28^ early afterdepolarizations,^29^ and field potential duration.^28^ These changes in the electrical substrate may lead to VA. Similarly, methamphetamine has been shown to alter the transcription and functions of cardiac ions channels, including the inhibition of transient outward potassium current, inward rectifying potassium current, and L-type calcium current.^30^ Electrocardiograms of methamphetamine users have shown higher rates of QTc prolongation, suggesting underlying changes in the electrical substrate that may lead to VA.^31^ Both cocaine and methamphetamine can cause enhanced catecholaminergic effects, myocardial oxidative stress, inflammation, necrosis, hypertrophy, collagen deposition, and fibrotic remodelling, all resulting in reduced systolic function and scar tissue that represent a structural substrate propitious to arrhythmias.^6^ These changes have been observed mostly in the setting of myocardial ischaemia^11^ but also in the absence of ischaemia. Besides the electrical and structural remodelling that could be induced by chronic cocaine or methamphetamine, rendering ventricular substrate prone to arrhythmogenesis, acute proarrhythmic consequences related to ion channel or catecholaminergic effects may have been operative in some cases. It is also possible that overdoses of these stimulants could acutely cause CA and sudden death as a final common pathway to multiple lethal mechanisms.^5^

It is worth emphasizing that these stimulants, especially methamphetamine use, were associated with comparable or even greater effect sizes in predicting VA in the current study than other common cardiovascular risk factors, including diabetes mellitus, hypertension, dyslipidemia, coronary artery disease, and other atherosclerotic cardiovascular diseases. Hence, avoidance of methamphetamines and cocaine should be an important target in strategies aimed at preventing VA and premature mortality. Our findings also support the recommendation to systematically investigate illicit drug use following CA as emphasized by the recent Lancet Commission on SCD.^32^ Furthermore, association of methamphetamine use with increased risk of VA and all-cause mortality raises questions about the safety of amphetamine used for disorders such as ADHD, narcolepsy, or obesity. Studies are needed to investigate the potential long-term risks associated with amphetamine in treated patients.

The risk of VA associated with cocaine use was significantly higher in Blacks and lower in Whites. This could be explained by the difference in the type of cocaine predominantly consumed in these groups. It has been shown that being Black may be associated with crack cocaine use, whereas being White has been associated with powder cocaine use.^33^ Powder cocaine is usually abused by snorting or by injecting into a vein, whereas crack cocaine is smoked. Smoking crack cocaine is more likely to be psychologically addictive and lead to chronic, heavy cocaine use than the predominant method for administering powder cocaine (snorting).^34^ Hence, more chronic and heavier cocaine use in Blacks could at least partly explain the apparent greater effect of cocaine on incident VA compared with other racial or ethnic groups. Unlike cocaine, methamphetamine use had a consistently higher risk of VA across all demographic groups. However, Blacks exhibited a substantially lower risk associated with methamphetamine, whereas Asians experienced a high risk. The reasons for these discrepancies remain unclear—a better understanding of the interactions between various sociocultural and behavioural factors and illicit drug use might shed some light on these racial and ethnic differences and reveal information pertinent to reducing risks among all people that use or are at risk of using these substances.

It is also remarkable that the impact of methamphetamine and cocaine on the incidence of VA was higher in younger people and females. This may suggest that the proarrhythmic properties of these stimulants are independent of aging. The observed higher predictive value for methamphetamine and cocaine in females compared with males deserves further investigation. The sex differences in particular might be due to ventricular structural and electrophysiological differences resulting from the impact of sex hormones during cell development, differences in autonomic function, differences in body size (such that the same amount of a drug leads to greater serum concentrations), or the effects of circulating sex hormones before and after puberty.^35^ Another potential explanation is the fact that a risk factor for a disease tends to have a higher attributable risk in the absence of competing risk factors.^36^ Hence, the lower prevalence of classical risk factors for VA such as coronary artery disease in younger individuals and females might explain why they have relatively higher risk of VA attributable to methamphetamine and cocaine use compared with older individuals and males.

Our study has several limitations. The use of administrative databases and diagnostic codes is prone to coding errors and incomplete documentation. However, our additional validation analyses of codes for cocaine and methamphetamine exhibited reasonable sensitivities and specificities. Furthermore, previous research has shown good accuracy of codes for methamphetamine and cocaine use as well as for VT, VF, and CA.^26,37–39^ This also points to a potential advantage of the current study as, due to common stigma, studies relying on self-reported use of these substances may be inaccurate.^40^ In addition, similar study design and statistical methods utilizing earlier years of similar datasets have resulted in conclusions reproduced in various other research settings.^36,41–44^ Because we only included individuals who presented to a healthcare setting, we mostly captured people with substance use that was severe enough to catch the attention of a healthcare provider. Hence, our findings might not be fully applicable to those with lower or rare consumption of cocaine and methamphetamine. However, this would not detract from the differential risks among these millions of patients with and without evidence of illicit stimulant use nor the differential magnitudes of risks relative to established cardiovascular co-morbidities within this same population. We recognize systemic bias may be operative in the current data base such that differential misclassification might favour statistically significant results among those coded as using stimulants (such as if those patients generally engaged in overall more healthcare utilization or if coding for stimulants was a marker of coding for all other diagnoses in general), but the negative control analyses suggested such differences were unlikely to explain our observations. In addition, sensitivity analyses revealed similar results when outcomes were constrained only to those with out-of-hospital cardiac arrest presenting to the emergency department or when examining mortality outside a healthcare facility, providing evidence the differences observed were not simply among those already admitted for some other disease. Finally, as with all observational studies, we cannot exclude residual or unmeasured confounding, limiting confident conclusions regarding causal inference. Despite these limitations, to our knowledge, this study is the first large longitudinal cohort study examining methamphetamine and cocaine use as predictors of incident VA and all-cause mortality. Furthermore, the study covered most of the California's adult population, which is one of the most diverse in the United States, allowing a robust analysis of sociodemographic interactions. Substance use and all other covariates were time-updated at each healthcare encounter, and death included as a competing risk for VA, providing for temporal assessments accounting for a competing risk of death (where use of the Comprehensive Death File assured that we included all deaths in California over the study period).

## Conclusion

Methamphetamine and cocaine use are each associated with a heightened risk of incident VA and all-cause mortality. The magnitude of the risk associated with these substances was similar to most well-established cardiovascular risk factors. Unlike many of those risk factors and with the caveat that physical addiction may limit ready cessation of these drugs, these drugs represent a modifiable risk factor under the immediate control of the user. These findings stress the importance of avoiding methamphetamine and cocaine to prevent VA and premature death. Recognizing and, in the future, better understanding the differences in the impact of these substances across demographic groups may enhance prevention strategies.

## Supplementary Material

ehaf282_Supplementary_Data

## Declarations

### Data Availability

The data analyzed are available with permission from the Department of Health Care Access and Information (HCAI) at https://hcai.ca.gov/data/request-data/.
