# Supplementary material for: Illicit stimulants and ventricular arrhythmias: a longitudinal cohort study
Source: Eur Heart J. 2025 May 7;46(37):3639–47. doi: 10.1093/eurheartj/ehaf282 (PMC12488323; doi:10.1093/eurheartj/ehaf282)
Supplement: ehaf282_Supplementary_Data [file ehaf282_supplementary_data.docx]

**APPENDIX**

**Illicit Stimulant Use and Ventricular Arrhythmias**

**Figure S1**. Participant selection


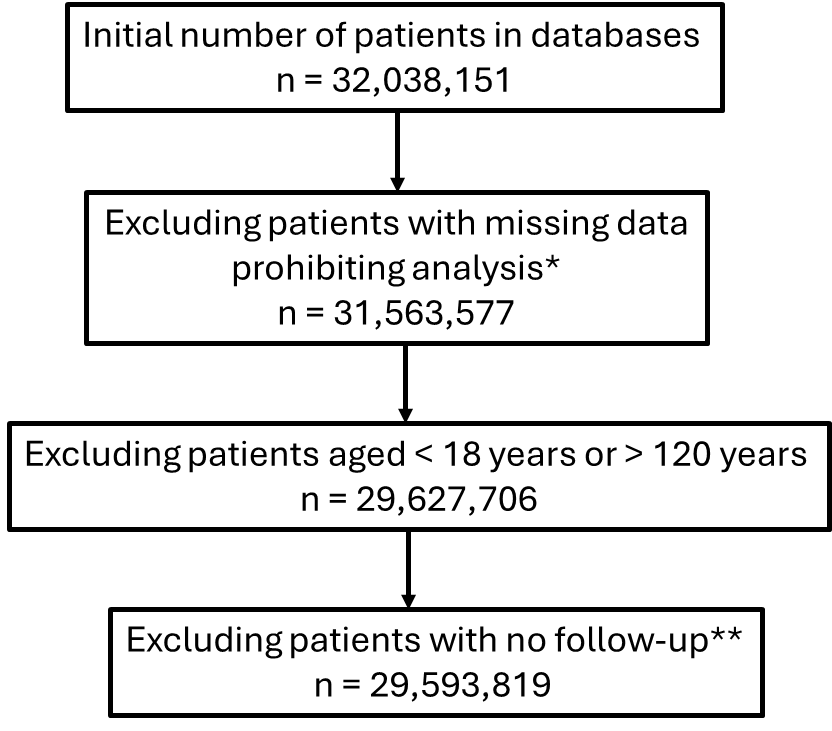


* Missing data on sex and race

** Patients who entered the cohort (first healthcare encounter) on the same day than their death or end of the study (December 31, 2019)

**Table S1**. CCS, ICD-9, and ICD-10 codes for diagnoses

| **Diagnoses** | **CCS** | **ICD-9** | **ICD-10** |
| --- | --- | --- | --- |
| Acromegaly |  | 253.0 | E22.0 |
| ADHD |  | 314.0 | F90 |
| Alcohol abuse |  | 291.x, 303.x, 303.00, 305.0, 790.3, 980.0 | F10.x, T51.x, R78.0 |
| Amphetamine use |  | 304.40, 304.41, 304.42, 304.43, 305.70, 305.71, 305.72, 305.73, 969.72 | F15.1x, F15.2x, F15.9x |
| Cannabis |  |  | F12.1x, F12.2x, F12.9x |
| Cardiac arrest |  | 427.5 | I46 |
| Chronic kidney disease |  | 585.x | N18.x |
| Chronic obstructive pulmonary disease |  | 491, 492, 496 | J41, J42, J43, J44 |
| Cocaine use |  | 304.20, 304.21, 304.22, 304.23, 305.60, 305.61, 305.62, 305.63, 970.81 | F14.1x, F14.2x, F14.9x |
| Coronary heart disease |  | 410.x to 414.x | I20.x to I25.x, Z95.1, Z95.5, Z98.61 |
| Diabetes mellitus | 49, 50 | 250.xx | E10.x to E13.x |
| Dyslipidemia | 53 | 272.0 to 272.5 | E78.x |
| Heart Failure | 108 | 428.x | I11.0, I13.0, I13.2, I50.x |
| HIV infection |  | 042 | B20 |
| Hypertension | 98, 99 | 401.x | I10.x to I16.x, I67.4 |
| Ischemic stroke |  | 433.x, 434.x, 436.x, 437.x, | I63.x, G46 |
| Myasthenia gravis |  | 358.0 | G70.0 |
| Narcolepsy |  | 347 | G47.4 |
| Obesity-related disorders |  | 278.00, 278.01, 278.03 | E66.0, E66.1, E66.2, E66.8, E66.9 |
| Opioid use |  | 304.00, 304.01, 304.02, 304.03, 304.70, 304.71, 304.72, 304.73, 305.50, 305.51, 305.52, 305.53, 965.00, 965.01, 965.02, 965.09, E850.0, E850.1, E850.2, E935.0, E935.1, E935.2 | F11.1x, F11.2x, F11.9x |
| Peripheral artery disease |  | 440.2x, 440.3x, 440.4x, 443.9 | I70.x, I71.x, I73.1, I73.9 |
| Presbycusis |  | 388.01 | H91.1x |
| Smoking |  | V15.82, 305.1, 649.0, 989.84 | Z72.0, F17.21, F17.20, Z87.891 |
| Ventricular tachycardia |  | 427.1 | I47.2 |
| Ventricular fibrillation |  | 427.41 | I49.01 |

ADHD: Attention-deficit/hyperactivity disorder; CCS: Clinical Classification Software; ICD-9: International Classification of Diseases, Ninth Revision; ICD-10: International Classification of Diseases, Tenth Revision

**Table S2. Consistency between diagnostic codes and documented substance use in medical records**

|  | **Methamphetamine** | | **Cocaine** | |
| --- | --- | --- | --- | --- |
|  | **Documented use** | **No documented use** | **Documented use** | **No documented use** |
| ICD code present | 97 | 3 | 100 | 0 |
| ICD code absent | 3 | 97 | 7 | 93 |

ICD code: International Classification of Diseases code

Documentation of substance use was determined by the review of patients’ electronic medical records.

**Table S3. Crude incidence rates of ventricular tachycardia, ventricular fibrillation, cardiac arrest, ventricular arrhythmias and all-cause mortality stratified by methamphetamine and cocaine uses**

| **Outcomes** | **Methamphetamine** | | | **Cocaine** | | |
| --- | --- | --- | --- | --- | --- | --- |
|  | **Non-users** | **Users** | **P value** | **Non-users** | **Users** | **P value** |
| Ventricular arrhythmias | 2.35 (2.34-2.35) | 4.56 (4.44-4.67) | <0.001 | 2.35 (2.34-2.36) | 5.02 (4.83-5.21) | <0.001 |
| Ventricular tachycardia | 1.19 (1.18-1.19) | 2.35 (2.27-2.44) | <0.001 | 1.19 (1.19-1.19) | 2.58 (1.45-2.72) | <0.001 |
| Ventricular fibrillation | 0.27 (0.27-0.27) | 0.54 (0.50-0.58) | <0.001 | 0.27 (0.27-0.28) | 0.66 (0.59-0.73) | <0.001 |
| Cardiac arrest | 1.26 (1.25-1.26) | 2.54 (2.46-2.63) | <0.001 | 1.26 (1.26-1.26) | 2.80 (2.66-2.94) | <0.001 |
| All-cause death | 10.84 (10.83-10.85) | 7.13 (6.99-7.28) | <0.001 | 10.82 (10.80-10.83) | 14.14 (13.81-14.47) | <0.001 |

Incidence rates are expressed in 1000 person-years (95% confidence interval)

**Table S4. Sensitivity analyses for the risk of cardiac arrest coded in the emergency department and out-of-hospital mortality associated with methamphetamine and cocaine uses**

|  | **Cardiac arrest coded in the emergency department** | | **Out-of-hospital mortality** | |
| --- | --- | --- | --- | --- |
|  | **HR (95% CI)** | **P value** | **HR (95% CI)** | **P value** |
| Substance use |  |  |  |  |
| Methamphetamine | 1.87 (1.76-1.97) | <0.001 | 1.52 (1.48-1.55) | <0.001 |
| Cocaine | 0.95 (0.87-1.04) | 0.25 | 1.72 (1.68-1.77) | <0.001 |

Hazard ratio with 95% confidence interval, adjusted for demographics (age, sex, race and ethnicity), insurance status, level of income, substance use (methamphetamine, cocaine, cannabis, opioid), tobacco smoking, alcohol abuse, and co-morbidities (hypertension, diabetes, dyslipidemia, heart failure, chronic kidney disease, coronary artery disease, peripheral artery disease, ischemic stroke).

**Table S5. Sensitivity analyses of the risk of ventricular arrhythmias associated with methamphetamine and cocaine uses after exclusion of patients with selected conditions**

|  | **Ventricular arrhythmias** | | | | | |
| --- | --- | --- | --- | --- | --- | --- |
|  | **Patients with obesity-related disorders excluded** | | **Patients with ADHD or narcolepsy excluded** | | **Patients with HIV excluded** | |
|  | **HR (95% CI)** | **P value** | **HR (95% CI)** | **P value** | **HR (95% CI)** | **P value** |
| Substance use |  |  |  |  |  |  |
| Methamphetamine | 1.91 (1.86-1.97) | <0.001 | 1.90 (1.85-1.95) | <0.001 | 1.90 (1.85-1.95) | <0.001 |
| Cocaine | 1.18 (1.13-1.23) | <0.001 | 1.15 (1.10-1.19) | <0.001 | 1.13 (1.09-1.18) | <0.001 |

ADHD: attention-deficit/hyperactivity disorder; CAD: coronary artery disease; CI: confidence interval; CKD: chronic kidney disease; HIV: human immunodeficiency virus; HR: hazard ratio; PAD: peripheral artery disease
Hazard ratio with 95% confidence interval, adjusted for demographics (age, sex, race and ethnicity), insurance status, level of income, substance use (methamphetamine, cocaine, cannabis, opioid), tobacco smoking, alcohol abuse, and co-morbidities (hypertension, diabetes, dyslipidemia, heart failure, chronic kidney disease, coronary artery disease, peripheral artery disease, ischemic stroke).

**Table S6. Baseline participants characteristics according to cocaine use, before and after propensity score matching for the outcome of ventricular arrhythmia**

|  | **Before propensity score matching** | | **After propensity score matching** | |
| --- | --- | --- | --- | --- |
|  | **No cocaine use**  **(n = 29,228,316)** | **Cocaine use**  **(n=285,333)** | **No cocaine use**  **(n=123,743)** | **Cocaine use**  **(n=123,743)** |
|  |  |  |  |  |
| Mean age (years) | 45.0 ± 19.4 | 40.5 ± 13.7 | 41.8 ± 14.4 | 41.4 ± 13.7 |
| Male (%) | 13,420,861 (45.9) | 192,940 (67.6) | 68,800 (55.6) | 68,622 (55.5) |
| Race and ethnicity (%) |  |  |  |  |
| Asian | 2,914,265 (10.0) | 6,800 (2.38) | 6,334 (5.1) | 6,203 (5.0) |
| Black | 2,098,413 (7.2) | 85,963 (30.1) | 23,837 (19.3) | 23,496 (19.0) |
| Hispanic | 7,689,561 (26.3) | 68,677 (24.1) | 30,340 (24.5) | 30,363 (24.5) |
| Native American | 105,240 (0.4) | 964 (0.3) | 598 (0.5) | 637 (0.5) |
| White | 15,140,106 (51.8) | 114,955 (40.3) | 57,625 (46.6) | 58,055 (46.9) |
| Other | 1,280,731 (4.4) | 7,974 (2.8) | 5,009 (4.1) | 4,989 (4.0) |
| Co-morbidities |  |  |  |  |
| Diabetes (%) | 2,007,272 (6.9) | 41,816 (14.7) | 16,095 (13.0) | 15,588 (12.6) |
| Hypertension (%) | 4,356,815 (14.9) | 95,511 (33.5) | 29,600 (23.9) | 29,786 (24.1) |
| Heart failure (%) | 437,973 (1.5) | 20,941 (7.3) | 8,443 (6.8) | 7,803 (6.3) |
| Dyslipidemia (%) | 2,089,348 (7.2) | 39,070 (13.7) | 15,096 (12.2) | 15,153 (12.3) |
| CKD (%) | 320,230 (1.1) | 14,773 (5.2) | 6,196 (5.0) | 5,800 (4.7) |
| CAD (%) | 974,053 (3.3) | 17,171 (6.0) | 8,160 (6.6) | 7,879 (6.4) |
| PAD (%) | 145,139 (0.5) | 2,119 (0.7) | 1,718 (1.4) | 1,444 (1.2) |
| Ischemic stroke (%) | 204,992 (0.7) | 7,188 (2.5) | 3,734 (3.0) | 3,303 (2.7) |

CAD: coronary artery disease; CKD: chronic kidney disease; PAD: peripheral artery disease

Baseline is the first healthcare encounter for non-users of cocaine and the first healthcare encounter with a diagnosis code for cocaine use for users

Cohorts matched based on age, sex, race, co-morbidities (hypertension, diabetes mellitus, heart failure, dyslipidemia, chronic kidney disease, coronary artery disease, peripheral artery disease, chronic obstructive pulmonary disease, and ischemic stroke)

**Table S7. Baseline participants characteristics according to methamphetamine use, before and after propensity score matching for the outcome of ventricular arrhythmia**

|  | **Before propensity score matching** | | **After propensity score matching** | |
| --- | --- | --- | --- | --- |
|  | **No methamphetamine use**  **(n = 28,832,451)** | **Methamphetamine use**  **(n=681,198)** | **No methamphetamine use**  **(n=325,892)** | **Methamphetamine use**  **(n=325,892)** |
|  |  |  |  |  |
| Mean age (years) | 45.2 ± 19.5 | 38.3 ± 12.9 | 41.3 ± 12.7 | 41.1 ± 12.5 |
| Male (%) | 13,191,952 (45.8) | 423,947 (62.2) | 171,045 (52.5) | 170,306 (52.3) |
| Race and ethnicity (%) |  |  |  |  |
| Asian | 2,904,292 (10.1) | 17,148 (2.5) | 15,456 (4.7) | 15,553 (4.8) |
| Black | 2,116,846 (7.3) | 65,701 (9.6) | 29,039 (8.9) | 28,811 (8.8) |
| Hispanic | 7,560,952 (26.2) | 201,784 (29.6) | 91,771 (28.2) | 91,636 (28.1) |
| Native American | 101,192 (0.4) | 5,067 (0.7) | 1,927 (0.6) | 1,937 (0.6) |
| White | 14,881,823 (51.6) | 371,661 (54.6) | 174,032 (53.4) | 174,328 (53.5) |
| Other | 1,267,331 (4.4) | 19,837 (2.9) | 13,667 (4.2) | 13,627 (4.2) |
| Co-morbidities |  |  |  |  |
| Diabetes (%) | 1,994,722 (6.9) | 83,850 (12.3) | 31,327 (9.6) | 30,840 (9.5) |
| Hypertension (%) | 4,337,654 (15.0) | 186,776 (27.4) | 55,570 (17.1) | 55,998 (17.2) |
| Heart failure (%) | 434,730 (1.5) | 40,939 (6.0) | 13,568 (4.2) | 12,874 (4.0) |
| Dyslipidemia (%) | 2,084,584 (7.2) | 74,175 (10.9) | 28,680 (8.8) | 28,776 (8.8) |
| CKD (%) | 319,997 (1.1) | 22,753 (3.3) | 8,595 (2.6) | 7,926 (2.4) |
| CAD (%) | 971,898 (3.4) | 26,558 (3.9) | 13,129 (4.0) | 13,228 (4.1) |
| PAD (%) | 144,822 (0.5) | 3,888 (0.6) | 2,861 (0.9) | 2,522 (0.8) |
| Ischemic stroke (%) | 204,123 (0.7) | 13,391 (2.0) | 5,614 (1.7) | 5,256 (1.6) |

CAD: coronary artery disease; CKD: chronic kidney disease; PAD: peripheral artery disease

Baseline is the first healthcare encounter for non-users of cocaine and the first healthcare encounter with a diagnosis code for cocaine use for users

Cohorts matched based on age, sex, race, co-morbidities (hypertension, diabetes mellitus, heart failure, dyslipidemia, chronic kidney disease, coronary artery disease, peripheral artery disease, chronic obstructive pulmonary disease, and ischemic stroke)

**Table S8. Sensitivity analyses of the risk of ventricular arrhythmias associated with methamphetamine and cocaine based on propensity matched cohorts**

|  | **Ventricular arrhythmias** | |
| --- | --- | --- |
|  | **HR (95% CI)** | **P value** |
| Methamphetamine | 1.18 (1.14-1.21) | <0.001 |
| Cocaine | 1.31 (1.26-1.37) | <0.001 |

Hazard ratio with 95% confidence interval
